# The instruments of Eurozone fiscal surveillance through the lens of the soft law/hard law dichotomy—Looking for a new approach

**DOI:** 10.1057/s41261-021-00161-5

**Published:** 2021-06-19

**Authors:** Paul Dermine

**Affiliations:** grid.15711.33 0000 0001 1960 4179 M.A (College of Europe), LL.M (NYU), PhD (Maastricht University/KULeuven), European University Institute, Max Weber Fellow; KULeuven Institute for European Law, Affiliated Member, Florence, Italy

**Keywords:** Fiscal surveillance, Eurozone, Soft law, Bindingness, Legal effects, Disconnect

## Abstract

The past decade has profoundly reshaped the fiscal governance system of the Eurozone. Supranational prerogatives vis-à-vis State budgets have been significantly expanded, thereby redefining the nature of Union action in the field of fiscal policy and transforming the dynamics between the Union and its Member States. In spite of its overhaul and the practical effects that Eurozone fiscal governance now produces on the ground, the paper shows that overall, this regulatory system still formally qualifies as soft law. This results in a deep disconnect between the form and substance of Eurozone fiscal surveillance in the Eurozone, which raises a number of constitutional challenges. The paper shows that the source of this disconnect is to be found in the strict apprehension of the hard law/soft law divide and the narrow understanding of bindingness attached to it, which currently prevails in the legal discipline, but no longer corresponds to the realities of the EU’s regulatory practice. From there on, the paper offers an alternative approach towards the distinction between hard and soft law, based on a renewed, more open and contextual, understanding of the concepts of bindingness and legal effects, which might reconcile the form and the reality of Eurozone fiscal governance nowadays.

## Introduction

It is now commonplace to classify the legal production of any public authority as either hard law or soft law. In the European Union (EU), this *summa divisio* is deeply ingrained in the mind of law-makers, judicial actors and EU lawyers more generally. Over the past decade, EU policy-making has been marked by an increasing level of diversity and complexity and atypical instruments of law-making have started proliferating. It is only natural that the Union had to adjust its integration methods and techniques to address the challenges it was confronted with. One might however doubt whether a strict dichotomy between hard and soft law can still appropriately reflect the reality of the EU’s regulatory practice today. The issue of the continued relevance of the ‘hard law/soft law’ divide is not a purely conceptual one. It has deep constitutional repercussions and affects cardinal principles of the EU legal order, such as access to justice, accountability and transparency.

Fiscal governance in the Eurozone paradigmatically embodies this challenge and the risks that it implies for the European polity and the EU legal order. It has indeed been marked by a transformation of the EU’s regulatory activity, a shift in the power dynamics between the Union and its Member States, which can but very uneasily be captured through the ‘hard law/soft law’ distinction, thereby putting essential constitutional guarantees under strain. This article seeks to investigate this disconnect, its causes, and the most prominent manifestations. It also offers a way out, by outlining an alternative approach which could reconcile the reality of fiscal governance in the Eurozone today, with the legal categories through which it might be understood.

This article is structured as follows. It first provides a short overview of the system of fiscal surveillance currently in place in the Eurozone (2.). It then shows that the formal characterization of this system as a soft law framework lies in stark contrast with the practical effects it produces on the ground and the concrete powers with which the EU is now endowed in the field, producing a disconnect which proves highly problematic from a constitutional perspective (3.). The rest of the article is devoted to outlining a way out of this conundrum (4). The article shows that this disconnect is primarily rooted in a particularly rigid approach towards the ‘hard law/soft law’ divide and an excessively strict understanding of the principles of bindingness and legal effects. It then moves to offer an alternative approach to these concepts, which might reconcile the reality and the formal apprehension of fiscal surveillance in the Eurozone today. In spite of its clear focus on fiscal governance in the Eurozone, this article also seeks to provide more general reflections about the evolution of the EU’s regulatory practices and their apprehension by law, applicable to a wider diversity of policy fields.

## Fiscal surveillance in the Eurozone in the post-crisis era

As is well-known, the Economic and Monetary Union (EMU) was established as an asymmetric system. The currency union was indeed set up without a parallel pooling of national sovereignties on fiscal matters. Under the pattern favored in Maastricht, budgetary powers and responsibilities remain firmly anchored at the state level, following a decentralized pattern and the EU’s role in the field of fiscal policy is limited to one of coordination and surveillance (Articles 5(1) and 121(1) TFEU). The EU was not endowed with the positive capacity and means to conduct a fiscal policy of its own (the ‘no fiscal union’ option) and was confined to a role of supervision, overseeing national budgets and their compliance with the fiscal policy rules collectively agreed upon (the so-called ‘Maastricht criteria’ on debt and deficit), so as to mitigate the potential adverse effects of decentralized economic policy-making in the currency union.^1^

At the beginning, the surveillance conducted was quite loose, open, and mindful of national budgetary autonomy.^2^ However, the Eurocrisis brought into the open the structural deficiencies of this initial system of fiscal governance. Fiscal policy rules turned out to be blatantly disregarded, without meaningful reaction from its supposed enforcers (the EU institutions and the markets) and with well-known consequences. The Eurocrisis put the reform of the EMU’s economic pillar high on the political agenda and the fiscal governance system of the Eurozone has been subject to a profound and continuous overhaul ever since.

The many reforms passed in the field over the past decade—examined more in-depth in the following paragraph—have contributed to dramatically changing the face of fiscal supervision in the Eurozone and brought about a new system of fiscal governance. In a nutshell, this system presents the following characteristics.^3^ Its substantive scope has been expanded and includes most relevant budgetary variables (debt, deficit, structural balance, expenditure levels, …), thereby enabling EU institutions to assess and question, all the core determinants of national fiscal systems. The procedures and mechanisms supporting the system have been substantially developed and hardened, bringing about a much more intense, intrusive, and constraining pattern of surveillance. Eurozone Member States today are subject to dense networks of reporting duties, monitoring processes, and review mechanisms which place them in a situation of constant interaction with and continued assessment and scrutiny by, the EU institutions with regard to their budgetary policy. The complex procedural architecture underlying today’s EU fiscal governance system inserts national authorities in a solid and adaptable framework geared towards continuous reason-giving, justification, accountability, and pre-commitment. Fiscal surveillance in the post-crisis era is also characterized by a much harder approach towards compliance inducement and enforcement. The punitive aspects of the regime have been extended and strengthened. New forms of incentives, starting with macroeconomic spending conditionalities, are increasingly relied upon. Finally, the institutional structure of EU fiscal governance was deeply reconfigured, leading to a reversal in the relationship (and power dynamics) between the EU and its Member States and contributing to further centralize and supranationalize fiscal surveillance in the Eurozone.

A decade of continuous reforms has turned Eurozone fiscal governance into a highly complex regulatory system. In a nutshell, it is constituted of the following key procedural components. First, there is an exceptional regime, which very much constitutes the apex of fiscal surveillance in the Eurozone, under which States receiving financial assistance (so-called programme countries) commit to comprehensive adjustment programs and are subject to close supranational monitoring and surveillance.^4^ Beyond this regime of ‘extreme’ economic governance, there is the ‘standard’ system of economic and budgetary coordination and surveillance, which applies indistinctly to all Member States and shall constitute the main focus of this analysis. The Stability and Growth Pact (SGP) constitutes the backbone of this system. At its core, one finds the preventive arm of the SGP.^5^ It consists of a yearly coordination cycle under which the fiscal policies and budgetary trajectories of the Member States are scrutinized and their compliance with the Union’s fiscal policy rules assessed. The process, which has since 2012 been incorporated into the broader framework of the European Semester,^6^ culminates in the Union addressing country-specific recommendations (CSR’s) to national authorities. Since 2014, the preventive arm of the SGP is complemented by a procedure, under which national authorities submit their draft budgets before they are voted on, so that their compliance with the Eurozone’s fiscal rulebook can be assessed *ex ante*.^7^ Under this procedure, the Commission issues opinions on draft budgets and can eventually force Eurozone states to amend their budgets in case of violation of the collective fiscal policy rules. The SGP also comprises a corrective arm,^8^ the so-called ‘excessive deficit procedure’ (EDP), which is to function as an emergency brake, by correcting the major fiscal slippages that the preventive arm did not manage to prevent. The EDP is a complex procedure, supported by harder elements and an elaborated regime of sanctions, which is designed to bring back transgressive states onto the path of fiscal virtue, through a mixture of binding decisions and recommendations. As a final, transversal remark, the new economic governance of the Eurozone now relies on a convoluted enforcement regime. Beyond the punitive mechanisms that support the harder prongs (possibility of financial sanctions, …),^9^ there is also the deeply ingrained gradation of the system. As the budgetary health of a particular Member State deteriorates or improves, it will enter or exit a new procedural phase under EU economic governance and the pressure on the national policy space will mount or decrease. Importantly, the new system increasingly relies on incentives of all sorting and most notably so-called ‘macroeconomic spending conditionalities’: State access to EU funding is made dependent on good compliance with the policy injunctions being addressed under fiscal governance.^10^ This move away from sanctions, towards incentives, was recently confirmed with the Recovery Package agreed in July 2020 by the Heads of State and Government. Access to the funds constituting the biggest common fiscal stimulus in the history of the EU will indeed be made conditional upon the orderly implementation of Union recommendations on economic and fiscal policy.^11^

## Traditional categories outpaced by practice—the paradox of fiscal surveillance

This section looks more closely at the key instruments that make up Eurozone fiscal governance. First, following a rather traditional approach, we analyze these instruments in the light of the classic distinction between hard law and soft law and find that most of these acts qualify as soft law. Second, we contrast this finding with the practical effects of those acts and the impact that they concretely have on national policy spaces. We observe a deep disconnect between the formal characterization of Eurozone fiscal governance as a soft law framework and its de facto ability to shape and align budgetary policies across the currency union. This disconnect, as we shall see, raises several serious constitutional issues and questions some of the most fundamental organizing principles of the EU polity.

### Formal characterization of the instruments

The current system of ‘standard’ fiscal governance in the Eurozone does rely on certain instruments which undoubtedly qualify as hard law. Under its various constitutive procedures,^12^ European institutions, starting with the Council of the EU, are indeed competent to adopt decisions, i.e. acts which produce clear binding legal effects vis-à-vis their addressees (Article 288(4) TFEU). That is however not the case for the most central elements of that regulatory system. Indeed, the substance of the policy injunctions addressed by the EU to national fiscal authorities is primarily contained in acts that are traditionally seen as belonging to the realm of soft law. That is, first and foremost, the CSR’s States receive every year under the Semester process. That is also the special recommendations that States placed under an EDP are addressed by the Commission and the Council.^13^ Finally, that is the opinions through which the Commission conducts its yearly assessments of national draft budgets.

*Prima facie*, these instruments only qualify as soft law.^14^ Their formal label indeed implies that they were not intended to produce binding legal effects. After all, Article 288(5) TFEU provides that ‘recommendations and opinions shall have no binding force’. This seems to be confirmed by the general tone of these instruments and the vocabulary relied upon, which tends to remain open and non-peremptory. An explanation often put forward is that the limited competences of the Union in the economic and fiscal field would not allow for more constraining interventions. Data moreover suggest that the guidance provided by Union institutions in these tools is not consistently complied with at the national level. For example, in 2019, the European Parliament considered that only 2% of the European Semester CSR’s had been fully implemented, whereas 40% led to some progress and the remaining 58% produced no or limited progress.^15^ Such compliance deficit does however not seem to be legally reacted to by the Union and the Commission has not been seen mobilizing its traditional means of enforcement (starting with infringement proceedings) in the field of fiscal governance. On a related note, most of these instruments are not immediately backed up by formal sanctions which, as a general rule, only become available at the end of the procedures concerned.

Interestingly, what we observe for the instruments through which fiscal governance is operated, equally holds for the acts containing the very fiscal policy rules in the light of which coordination and supervision are conducted. The founding rules of the Eurozone’s fiscal rulebook are contained directly in the Treaties or in secondary law. But there has been an increasing tendency, within the EU institutions, to further specify and develop these rules and their interpretation, in post-legislative guidance (such as communications, guidelines or vade-mecums), that is instruments that still qualify as soft law.^16^

### The practical effects of fiscal surveillance

A closer look into the concrete impact of Eurozone fiscal governance on the ground shows us that its effects are far from the informative, preparatory, interpretative or formal/informal steering effects traditionally associated with soft law.^17^ This regulatory system indeed shapes and constrains national policy spaces and guides fiscal authorities throughout the Eurozone in a way that soft law is not supposed to or at least, was not designed to. To the informed observer, fiscal governance in the post-crisis era appears much harder than it is formally supposed to be.

Our view is that fiscal and economic governance in the post-crisis era has taken a critical ‘harmonizing’ turn. This means that this governance system is no longer limited, as it was in pre-crisis times, to the collective pursuit of a shared economic project and the attainment of common policy objectives through a diversity of structures, tools and approaches. It is now driven towards the joint implementation of a certain type of economic and fiscal policy (constituted by both specific policy objectives and the particular means to attain them) and hence pursues far-reaching substantive convergence^18^ by seeking to erase, or at least reduce, structural differences between the Member States. The ultimate aim is harmonization, alignment, i.e. the ordering of national policies in the economic and fiscal field along the lines of a homogenous model set at the supranational level.^19^ The model itself is not carved in stone and does evolve alongside the wider macroeconomic environment and political and ideological developments. What remains, however, is the EU’s ability to diffuse such model throughout the Union, following a harmonizing template. Space constraints prevent us from providing comprehensive analyses of instances of such harmonization. The recent evolution of the structure of national taxation systems in the Eurozone, the overall reduction of the tax wedge on labor and the trajectory of public expenditures across Europe however provide multiple illustrations of such ongoing alignment.^20^

These harmonizing effects are, admittedly, less immediately perceivable than in areas where classic harmonization is at play, such as the internal market. This is best explained by the enduring peculiarities of fiscal governance as a regulatory system. Fiscal governance in the Eurozone indeed embodies an unconventional form of policy integration which, in many regards, breaks away with the traditional methods of EU law and European integration. First, it relies on a looser understanding of compliance. Absolute compliance with its injunctions is not sought by the European Commission. This is also suggested its reaction to the low implementation rate of Semester CSR’s.^21^ The system is primarily about national endorsement of the overall fiscal and economic trajectory outlined by the Union. This is the reason why the numerous fiscal policy rules on which the system relies do not act as absolutes, but play a primarily deliberative role, providing a discussion platform and drawing certain red lines framing upcoming debates.^22^ Second, fiscal governance is also characterized by an unprecedented level of country-specificity and differentiation. This is a system under which general rules are translated into national targets, where programming plays a key role and where governance is increasingly conducted in a bilateral manner. This is also a variable-geometry system, characterized by great adjustability (and thereby asymmetry), where the pressure and constraint exercised on national authorities and the associated encroachments on national sovereignty, depend on the fiscal and macroeconomic health of States and the risks they pose to the currency union.^23^ Finally, fiscal governance constitutes a regulatory system where formal constraints (in the form of sanctions or legal actions) remain ultimately available, but are only very rarely relied upon in practice.^24^ The system is instead much more decisively geared by a diversified set of explicit or implicit political, financial, or administrative pressures. The post-crisis era has indeed seen emerging a regime of macroeconomic pressures,^25^ which exercises structuring effects on state budgets and national fiscal policies, without formal sanctions being de facto applied, or the intervention of a judge being available.

Altogether, the post-crisis era thus brought about, with Eurozone fiscal governance, a new way to drive integration and a new type of institutional and policy dynamics between the Union and the States. This system is inherently paradoxical: in spite of its informal nature and the ‘soft’ label of most of its outputs, it exercises strong harmonizing pressures on state authorities and significantly shapes and constrains national fiscal policies. The Union, if it is in most cases still not in a position to formally impose fiscal change at the national level, has gained additional tools and can now rely on an efficient governance framework to more or less forcefully induce it. This state of affairs signals a clear disconnect between the form and the substance of fiscal governance, between the legal characterization of its key instruments and their practical effects on the ground, between its allegedly ‘soft’ nature and its much ‘harder’ presence.

### A disconnect between the formal nature of fiscal surveillance and its practical effects and its constitutional dangers

There is thus a profound disconnect between the reality of Eurozone fiscal governance and the formal characterization of its key instruments. Traditional categories and conceptual tools on which EU law has long relied seem to have progressively fallen out of sync with the Union’s evolving normative landscape and regulatory practice and no longer account for what is actually unfolding on the ground. The phenomenon is particularly pertinent in the field of fiscal governance, to which this article is devoted. One should not lose sight of the fact that it however is of a wider, structural magnitude and concerns policy fields as diverse as banking and financial law, migration policy and external relations.^26^

This disconnect is profoundly disturbing. It raises a number of serious constitutional issues,^27^ which question the soundness of the foundations of the Eurozone’s new fiscal governance and its compliance with some of the governing principles of the EU polity.

The first issue is one of openness and transparency. The formal characterization of Eurozone fiscal governance in the post-crisis era as a soft law framework contributes to significantly downplaying the nature of that governance system, the influence it exercises on the Member States and the new division of powers that prevails in the field of fiscal policy. The EU’s continued reliance on the language and tools of the Open Method of Coordination (OMC) is as misleading as it is dangerous. It gives an erroneous impression of continuity between the pre-crisis and post-crisis eras and contributes to conceal the top-down and harmonizing dynamics that now characterize fiscal governance. The EU’s failure to formally embrace its new role and influence under the post-crisis system results in a dissonance between the discourse and the facts, the form and the substance of fiscal coordination and surveillance today in the EU. It has also led to a general blurring of the accountability lines in the field of fiscal and budgetary policy and it has brought about a configuration in which it is increasingly difficult for both citizens and institutions exercising review to attribute authorship and allocate responsibility for specific policy reforms.^28^ To borrow an expression forged by Tridimas,^29^ post-crisis fiscal governance in the Eurozone opens multiple spaces of ‘constitutional uncertainty’, which concerns the authorship of policy initiatives and the effects they bring about.

In direct relation to that first issue, there is also a problem of due consideration for the logic of separation of powers and of compliance with the competence allocation system that is to prevail in the EU polity. The ‘soft law’ label indeed enables Union institutions to expand their scope of action and penetrate policy spaces to order them, with only little regard for their own (limited) competences and the retained powers of national authorities.^30^

Another worrying issue is that these instruments that formally qualify as soft law cannot be challenged or contested in court by those who might have an interest in doing so (the Member States as direct addressees, interest groups or citizens whose position might be affected, …). For most of the output produced by the new fiscal governance system of the Eurozone, judicial review will remain unavailable. That means that in many instances, the act which constitutes the legal source of certain specific reforms implemented at the national level will evade any form of legal scrutiny. The reasons for such accountability gap are manifold, but primarily relate to the rules that govern the jurisdiction of the Court of Justice and the admissibility of cases brought before it. For example, actions for annulment under Article 263 TFEU can only be brought against acts deemed ‘challengeable’, a notion the Court interprets rather restrictively as only including acts producing binding legal effects (see *infra*). Naturally, soft law instruments such as CSR’s or Commission opinions do not satisfy this condition. Moreover, rules of standing before the Court of Justice are known to be particularly strict, especially for private applicants.^31^ As a result, the far-reaching prerogatives that the EU enjoys under the new fiscal governance system of the Eurozone and its strong ability to shape national policies, are not matched with appropriate channels of external review.

The formalization of fiscal governance as soft law not only complicates its law-based contestation in court, but also limits the possibility for political debate in representative fora. In general, soft law production in the EU system is primarily the business of executive bodies (the European Commission, the Council, EU agencies, …).^32^ Eurozone fiscal governance certainly is no exception. Such executive dominance^33^ implies that the intervention of the European Parliament, the EU’s only organ of direct citizen representation, will be, for most outputs of fiscal governance, limited to a minimum.^34^ The highly problematic nature of executive dominance and limited parliamentary involvement in the Union’s fiscal and economic affairs have already been analyzed in great length elsewhere.^35^ Let us simply recall here that the choices made and orientations favored under the various procedures that make up the Eurozone’s fiscal governance system are not purely technocratic ones that can be delegated in full to the executive branches of our institutional system. They have significant redistributive effects and are thus deeply political and value-based. Both democratic theory and European history teach us that such choices are best forged through the transparency and contestation that parliamentary assemblies embody, rather than behind the closed doors of an executive organ.

Last but not least, the fact that the material rules of the Eurozone’s fiscal rulebook are partly contained in soft law instruments also creates constitutional problems of its own. The proclaimed aim of post-legislative guidance is generally to clarify rules contained in the legislation, detail the interpretations the administration will favor and the ways it intends to use the flexibility and margins of maneuver it is granted by the texts, thereby increasing the transparency and predictability of the entire regulatory system.^36^ To a certain extent, fiscal post-legislative guidance, starting with the Commission’s Vade-Mecum on the SGP, does contribute to these goals. However, these instruments being formally soft, neither the Commission nor the Council consider themselves bound by the specifications made therein.^37^ Practice reveals inconsistencies and recurrent deviations from pre-established rules, which are most often not accounted for by the institutions in charge.^38^ The liberties hence taken by the Union institutions and the very contingent application of their own rules, leave a strong impression of discretion (if not arbitrariness) and unfairness between States,^39^ which is constitutionally problematic in a regulatory system which, as any other in the EU, is supposed to be governed by the principle of the rule of law.

Eurozone fiscal governance in the post-crisis era, as an informal and hybrid regulatory system contributes to disrupting the traditional ways and methods of EU law. The profound disconnect between the formal nature of this governance framework and the de facto pressure it exercises on national policy spaces, best embodies this disruption. This section shows how problematic this disconnect is from a constitutional perspective. Not only does it produce considerable constitutional uncertainty, but it also imperils key constitutional principles of the EU legal order, from effective judicial protection to conferral, openness and transparency. In essence, this disconnect reveals how the traditional understanding of EU law has progressively fallen out of sync with the EU’s evolving normative landscape and regulatory practice. If we want to reconnect Eurozone fiscal governance and, by extension, the other governance systems affected, with the constitutional code of the Union, it is urgent to reconsider some essential concepts and distinctions of our discipline, starting with the *summa divisio* between hard law and soft law. The next section aims at initiating such reflection, by taking fiscal governance as a starting point.

## Reconnecting the form and substance of fiscal surveillance in the Eurozone—Towards a new approach

### The source of the disconnect: an overly restrictive approach towards bindingness

Fiscal governance in the Eurozone is currently characterized by a constitutionally problematic disconnect, a misalignment between its formal characterization as a soft law governance framework and the practical, much harder, effects it deploys on the ground. This is in our view best explained by the disruptive effects fiscal governance has on the ways and methods through which European integration is traditionally carried out and the inability of EU law and EU lawyers so far to meaningfully apprehend these changes, so as to bridge the gap between form and substance. Classic and rigorist understanding of central concepts of our discipline, such as those of hard law, soft law, legal effects or bindingness, best explain why EU law has so far failed in grasping the evolution of the EU’s regulatory practice, the proliferation of hybrid policy instruments, the growing informalization of its governance structures and the diversification of its channels of enforcement. In essence, concepts, together with judicial interpretation, have fallen out of sync with reality and the felt impact of fiscal governance and the new integration mode it embodies, on the ground. If we ever want to reconcile the form and substance, the theory and practice of fiscal governance, there is an urgent need to revisit and reconstruct essential concepts of our discipline.

At the core of this enterprise, lies of course the conceptual distinction between hard law and soft law and the related notions of legal effects and constraint. The previous section has shown the progressive irrelevance of the sharp dichotomy traditionally drawn between hard and soft law; a dichotomy which over time fell out of sync with the realities of EU integration and the diversity and flexibility of the EU’s regulatory practice. It is our view that this *summa divisio* must be reconsidered and reinterpreted, so as to reconnect the legal characterization of Eurozone fiscal governance’s key policy instruments with the truth of the governance system they belong to and their concrete effects on the ground.

The concept of soft law is a complex one. Not only because of its elusiveness, but also because it covers a wide variety of institutional and regulatory practices. The concept has been the object of a very rich literature and many proposals have been made on how to measure the normative strength of legal instruments and determine their belonging to either hard or soft law.^40^ The European Court of Justice has not remained fully hermetic to the phenomenon of soft law and has, for a while, recognized that EU soft law does produce certain legal effects.^41^ The Court has also progressively developed a method to delineate the realms of hard law and soft law, at the center of which lies the concept of binding legal effects.^42^ A first key element in the Court’s method is the ‘substance over form’ approach, according to which it is the substance of the act or instrument at stake, rather than its form (or its label), which should determine its softness/hardness. Secondly, the Court has developed an approach towards bindingness characterized by its rigor and its positivism.^43^ The existence of binding legal effects is, following a classic formula, to be assessed, in the light of the wording and context of the act, its substance and the intention of its author. Practice reveals that this test is applied rather restrictively. What transpires from the Court’s case-law of the past two decades is that bindingness is, in a very classical way, closely connected to the possibility of coercion, i.e. the possibility for the act’s author to directly enforce and sanction the addressee which would have failed to comply with one of its prescriptions. Under such vision, the existence of direct enforcement channels and sanctions is determining.^44^

In our view, this strict apprehension of the hard law/soft law divide based on a narrow understanding of bindingness is overly restrictive and no longer corresponds to the realities of the EU’s regulatory practice. It precisely produces the kind of disconnect and misalignment that we have identified and denounced in the field of fiscal governance in the past section.

### Towards a renewed approach to the ‘hard law/soft law’ divide

Taking stock of the current stalemate, this section attempts to outline an alternative approach to the distinction between hard and soft law, based on a more open and contextual understanding of the concepts of bindingness and legal effects,^45^ which better suits the reality of Eurozone fiscal governance in the post-crisis era.^46^ We intend to do so by relying on one main source of inspiration: the opinion of AG Bobek in *Belgium v Commission*, a case which primarily concerned the legal status of an EU recommendation on online gambling and its reviewability under Article 263 TFEU.^47^

The key problem with soft law, which now proliferates in most fields of Union action, is that it challenges traditional legal categories and, in Bobek’s words, ‘does not easily fit within the binary, black and white distinction between binding and non-binding legal effects’.^48^ EU ‘soft law’ instruments may not be deemed binding in the classical sense, but they do serve a normative ambition and ‘generate considerable legal effects [both at the EU and at the national level], in the sense of inducing certain behavior and modifying normative reality’.^49^ Acts such as EU recommendations serve a quasi-legislative function by generating parallel sets of rules and can therefore be used to circumvent traditional decision-making channels and ‘shape the range of conceivable (acceptable) normative solutions for the future’.^50^ Still speaking of EU recommendations, Bobek revealingly concludes:

‘The point of recommendations is to induce compliance. Imagine a Member State which, having acted in good faith and in the spirit of sincere and loyal cooperation, has transposed a recommendation into the national law. By an act of national legislation, that Member State established obligations for individuals on the national level. Now, if that national legislation is challenged before the national courts, it would be somewhat peculiar to refuse the review of what constitutes the substantive basis of that national legislation, namely, the EU recommendation, with the somewhat formalistic excuse that what created those obligations was national legislation, not an EU law instrument and that the Member State did so purely of its own volition’.^51^

Because EU law, as interpreted by the Court of Justice, still relies on a narrow and positivist understanding of bindingness and overlooks the significance of soft law, our rules have progressively fallen ‘out of sync with the evolution of the EU’s normative landscape’.^52^ Because of an excessively restrictive yardstick of binding legal force, instruments of fiscal governance which have significant legal effects evade review and operate under the radars of EU law.^53^

Ways out of this conundrum do exist however. Reconnecting our legal structures with the evolving realities of Eurozone governance requires a more fluid understanding of the divide between hard and soft law, based on a much more open and contextual understanding of bindingness, structured around the existence of legal effects.

Assessing the existence of reviewable law should come down to a simple question: ‘could I, as a reasonable addressee, infer from the content, aim, general scheme and the overall context of a recommendation or, more generally, of a soft law instrument, that I am expected to do something?’^54^ The alternative test put forward is to be focused on the context and the content of the measure at issue, rather than on its purely formal wording, its label, or the availability of direct coercion mechanisms. In line with Bobek, 55 we envision this test as revolving around three cumulative sets of factors, of equal value. First, the degree of formalization and definitiveness of the measure at stake, through which the likelihood that the act will be perceived as producing legal effects is to be gauged. Second, the content and overall purpose of the act is to be reviewed. Of key importance will be the level of precision and prescription of the commitments and measures contained in the act and its underlying harmonizing purpose. Finally, compliance-inducing mechanisms and enforcement should be considered. A broad perspective is commended, that goes beyond traditional coercion methods (such as sanctions or legal enforcement), to embrace the diversity of direct and indirect compliance mechanisms, both structural and institutional.

### Fiscal governance under this new light

How might this alternative approach play out in the concrete case of Eurozone fiscal surveillance? The following paragraphs show that under this new light, one might gain a different and more truthful understanding of fiscal governance and its practical effects.

Taking the first criterion into account, namely the form of the act and its degree of definitiveness, we observe strong variations, but note that the key outputs of Eurozone fiscal governance display a fair level of formalization and take the form of legal acts.^56^ Moreover, most of the procedures they constitute the culmination of, are quite long, complex and elaborated. Eurozone fiscal governance is characterized by its continued nature. Organized along cycles (best epitomized by the European Semester process), it consists of a constant back-and-forth between the Union and its Member States. At the EU level, these procedures mobilize the entire EU institutional apparatus. As an example, CSRs are adopted by the Council on the basis of a Commission recommendation (following the ‘comply or explain’ rule). They are however discussed first and they are the subject of resolutions by the European Parliament in the framework of the Economic Dialogue. The European Council intervenes indirectly via its conclusions and no less than four Council committees contribute to shaping their content.

Through the second criterion, which relates to the content and purpose of the measures at issue, we focus on the level of precision and prescription of the commitments under scrutiny and their harmonizing purpose, as a key indicator of the legal effects produced. While a case-by-case analysis will always remain necessary, a few general observations can be made. First, certain categories of instruments remain fundamentally generic and abstract. This is most prominently the case for the Annual Growth Survey and the recommendations on the economic policy of the euro area. However crucial these might be in shaping the priorities, discourses, and cognitive structures of fiscal governance, they merely serve a programmatic purpose and do not produce many legal effects. But they are further materialized by other EU acts, of a country-specific nature, which, quite logically, display a higher level of precision and specificity and have a more authoritative tone. Again, nuance is essential. A deep look, for example, into the substance of CSRs reveals that, within one single set of recommendations, very detailed and concrete commitments, leaving the national authorities with only a handful of possible implementation options (thereby contributing to the harmonizing dynamics described in the above), can stand next to much more generic duties, barely curbing national margins of maneuver. For example, in 2019, Finland was recommended both to ‘strengthen the monitoring of household debt including by setting up a credit registry system’ and to ‘improve incentives to accept work’.^57^ In a similar fashion, in 2018, Italy was recommended to ‘reduce the length of civil trials at all instances by enforcing and streamlining procedural rules’ and to ‘shift taxation away from labor’.^58^ While under the EDP Portugal had been recommended to ‘adopt permanent consolidation measures worth at least 2% of GDP in view of attaining a headline deficit of 4% GDP in 2014’ and, in doing so, ‘to aim at streamlining and modernizing the public administration, addressing redundancies across the public sector functions and entities, improving the sustainability of the pension system’.^59^

The third criterion pertains to enforcement and comprehensively assesses the existence and strength of all mechanisms—formal or informal, procedural or substantive, legal or political, punitive or incentive-based—that are in place to induce compliance with the injunction contained in the instrument at stake. It essentially comes down to a simple question: considering the overall context around the act, will its addressee feel expected or compelled to act in a certain manner and pass new measures? This factor is in part subjective, as it also takes into account the perception of the addressee.^60^ As a general rule for Eurozone fiscal governance, note that the Member States’ duty to follow up on and comply with, the various injunctions and recommendations addressed to them in the framework of fiscal governance is most often explicitly enshrined in the relevant legislation.^61^ Coming to the availability of compliance-inducing mechanisms, we have observed a significant reconfiguration over the past decade, as the core outputs of Eurozone economic governance, even though still not formally binding, are today backed up by a wide and diverse set of mechanisms: the disciplining effect of the markets, financial sanctions, spending conditionality, financial incentives, ‘procedural’ sanctions, conclusion of ‘reform agreements’ with the EU, to mention a few. The conjunction of these elements strongly suggests that, in the field of Eurozone economic governance, national compliance with EU instruments is normatively expected and that follow-up via national implementing measures is awaited and encouraged, especially for countries in critical economic or fiscal condition. Take the case of Semester CSR’s. Even though they are not directly backed by formal sanctions, a Member State’s track record under the European Semester and the implementation of the CSR’s addressed to it in that context are important elements that EU institutions will rely on when contemplating degrading its overall economic and fiscal status under the economic governance regime, by subjecting it to ‘harder’ prongs of that regime, where it will be directly exposed to financial sanctions and an intensification of market pressures.^62^ It should be remembered that compliance with the CSR’s also conditions the access to a set of financial and procedural resources. Strong ties have already been established between European economic governance and cohesion policy, via ‘macroeconomic spending conditionality’. Crucially, this logic has been dramatically expanded under the Recovery Package, as the national recovery and resilience plans against the implementation of which Member States will receive funding, will be primarily built upon the recommendations they are addressed under the Semester. Finally, structural and processual elements also play an important part in inducing compliance. The opinions the Commission issues on draft budgetary plans under Regulation No. 473/2013 constitute another useful example. Under the Treaty system, opinions, just like recommendations, lack binding effect (Article 288(5) TFEU). However, under certain circumstances, these opinions will be perceived as inducing compliance, especially when the Commission considers that a draft budget implies particularly serious non-compliance or, to a lesser extent, risks of non-compliance. The main reason is that there are strong connections between the action of the EU Commission *ex ante* under Regulation No. 473/2013 and the decisions undertaken later by the EU institutions under both the preventive and corrective arms of the SGP. The recent Italian case is a clear example of these ties, as it suggests that negative opinions of the European Commission, if not appropriately acted upon by national authorities, are most likely to prompt the opening of an excessive deficit procedure under Article 126 TFEU, with all the far-reaching consequences that it entails for the State concerned. When dealing with these instruments, one should also not overlook their communicative power: negative opinions, or opinions pointing at risks of non-compliance, also act as a signal to the markets, which they seek to alert about certain worrying fiscal trends and the reaction of which is therefore reckoned upon, if not expected. The recent Italian case is a good illustration of this: the Commission’s negative opinions on the Italian DBP clearly alerted the markets and bond yields started to soar.^63^

Seen in this new light, we might gain a different and more truthful apprehension of Eurozone fiscal governance. Under this test, many outputs of this system would still qualify as soft law. Some of them, however, i.e., those that effectively prompt compliance at the national level and serve a quasi-legislative function, would finally evade this label, as the reality and significance of the legal effects they produce and their de facto hardness, would finally be embraced. This would greatly contribute to correcting the disconnect identified *supra*, reconciling the reality of fiscal surveillance in the Eurozone with its formal apprehension. It would also consolidate the constitutional foundations of that governance system and address many of the concerns expressed in the above in terms of consistency, transparency, responsibility-allocation, reviewability and contestability.

## Conclusion

Eurozone fiscal governance in the post-crisis era is characterized by a deep disconnect between its formal characterization as a ‘soft’ governance framework and the much harder effects that it produces in practice and the harmonizing dynamics it concretely sets in motion. This disconnect raises, as we saw, several fundamental constitutional issues and challenges some of the key organizing principles of the EU polity and the Union legal order. Our enquiry showed that this disconnect is best explained by the excessively rigid understanding of the soft/hard law divide that still prevails under EU law and a narrow construction of the concepts of bindingness and legal effects. For that matter, post-crisis fiscal governance stands as a paradigmatic example of law being outpaced by practice. It also stands as a wake-up call, emphasizing the necessity that EU lawyers revisit some of the essential concepts that govern their discipline. From there on, this paper has sought to offer an alternative approach to the soft/hard law divide, based on a much more open and contextual understanding of bindingness and legal effects. We saw that alternatives exist and might contribute to addressing this troubling disconnect between the form and the substance of Eurozone fiscal governance today, thereby reconnecting this central governance system of the Union with the constitutional values that found the European legal order.

## Footnotes

1 H. Hofmann, K. Pantazatou, G. Zaccaroni (eds), The Metamorphosis of the European Economic Constitution. Cheltenham: Edward Elgar, 82–105.

2 Maher, I. (2007), ‘Economic Governance: Hybridity, Accountability and Control’. Columbia Journal of European Law 13(3): 679–704.

3 See Dermine, P. (2018), Economic Governance in a Post-Crisis Era—A Conceptual Appraisal. European Papers 3(1): 281–306.

4 See, most notably, Article 13 of the Treaty establishing the European Stability Mechanism and Regulation No. 472/2013 of the European Parliament and of the Council on the strengthening of economic and budgetary surveillance of Member States in the Euro area experiencing or threatened with serious difficulties with respect to their financial stability, *OJ* L 140, 27.5.2013, pp. 1–14.

5 Article 121 TFEU and Council Regulation (EC) No. 1466/97 on the strengthening of the surveillance of budgetary positions and the surveillance and coordination of economic policies, *OJ* L 209, 2.8.97, p. 1–13. The Regulation was reorganized by Six-Pack Regulation (EU) No. 1175/2011 of the European Parliament and of the Council, *OJ* L 306, 23.11.2011, pp. 12–31.

6 See new Article 2-a of Regulation No. 1466/97.

7 Regulation (EU) No. 473/2013 of the European Parliament and of the Council on common provisions for monitoring and assessing draft budgetary plans and ensuring the correction of excessive deficits of the euro area Member States, *OJ* L 140, 27.5.2013, p. 11–25**.** Unlike the SGP, which applies to all EU Member States (with some Eurozone-specific aspects), this new procedure only applies to countries which are part of the currency union.

8 See Article 126 TFEU and Council Regulation (EC) No 1467/97 on speeding up and clarifying the implementation of the excessive deficit procedure, *OJ* L 209, 2.8.97, p. 6–13. The Regulation was reorganized by Six-Pack Regulation (EU) No. 1177/2011 of the Council, *OJ* L 306, 23.11.2011, pp. 33–38.

9 See Regulations (EU) No. 1173/2011 of the European Parliament and of the Council on the effective enforcement of budgetary surveillance in the Euro area, *OJ* L 306, 23.11.2011, p. 1–6.

10 Most notably, see Art. 23 of Regulation No. 1303/2013 of the European Parliament and the Council of 17 December 2013 (‘Common Provisions Regulation’), *OJ* L 347, 20.12.2013, pp. 320 ff.

11 European Council (2020) ‘European Council conclusions, 17–21 July 2020’, § 69; Regulations (EU) No. 2021/241 of the European Parliament and of the Council establishing the Recovery and Resilience Facility, *OJ* L157, 18.2.2021, p. 17–75; Articles 10, 19(3)(b), 24. On this aspect, see Dermine, P. (2020), ‘The EU’s Response to the COVID-19 Crisis and the Trajectory of Fiscal Integration in Europe—Between Continuity and Rupture’. Legal Issues of Economic Integration 47(4): 350–352.

12 In the framework of the EDP, see Article 126(6) on the existence of an excessive deficit, 126(9) on failure to comply, 126(11) on sanctions and 126(12) TFEU on the abrogation of the EDP. See also Articles 5–7 of Regulation No. 1173/2011 and Article 23(9) of Regulation No. 1303/2013 on sanctions. In the framework of the preventive arm of the SGP, see Article 6(2)(4 and 5) of Regulation No. 1466/97 on failure to take appropriate action addressing a significant deviation and Articles 4 and 8 of Regulation No. 1173/2011 on sanctions. In the framework of the enhanced surveillance procedure and extreme economic governance, see Article 7 on the macroeconomic adjustment program. Under the recently adopted Recovery and Resilience Facility, decisions will be adopted at each key stage of the process, starting with the approval of the national recovery and resilience plans (Article 20 of the Regulation No. 2021/241) and authorzation of the disbursement (Article 24).

13 See Article 126(7) TFEU. Adopted ‘with a view to bringing the deficit situation to an end within a given period’, these recommendations generally set the deadline by which the excessive deficit ought to be corrected, the core fiscal targets that should be reached and the main reform avenues through which correction is to be achieved.

14 Soft law is an intricate concept, whose definition greatly varies across the literature. In the EU context, this definition by Senden seems particularly appropriate: soft law consists in ‘rules of conduct that are laid down in instruments which have not been attributed legally binding force as such, but nevertheless may have certain (indirect) legal effects and that are aimed at and may produce practical effects’ (Senden, L. (2004), Soft Law in European Community Law. Oxford: Hart, 112). Alternatively, Snyder defines soft law as ‘commitments which are more than policy statements but less than law in its strict sense. They all have in common, without being binding as a matter of law, a certain proximity to the law or a certain legal relevance’ (Snyder, F. (1994), Soft Law and Institutional Practice in the European Community. In: S. Martin (ed.), Sources and Categories of European Union Law, Baden-Baden: Nomos, 198.

15 See European Parliament, Implementation of the 2019 Country-Specific Recommendations, 20 April 2020, PE 624.400.

16 The Commission has taken a leading role in that regard. See for example the 2015 Commission Communication on Flexibility. See also the extensive Vade Mecum on the Stability and Growth Pact, which the Directorate-General for Economic and Financial Affairs publishes every year since 2013 (DG ECFIN, Vade Mecum on the Stability and Growth Pact, European Economy Institutional Paper No. 101, April 2019).

17 On the functions served by soft law, see Senden, L., *supra* n. 15, 109–218.

18 See, in this regard, Scharpf, F. (2016), Forced Structural Convergence in the Eurozone—Or a Differentiated European Monetary Community, MPlfG Discussion Papers, No. 16/15.

19 Along these lines, see Martinez-Yanez, N. (2016), Rethinking the role of employment and social policy coordination competences in a deeper economic union. European Labour Law Journal 7(4): 523–547.

20 Fitoussi, J.-P., Saraceno, F. (2013). European Economic Governance: The Berlin-Washington Consensus. Cambridge Journal of Economics 37(3): 479–496; Lütz, S., Kranke, M. (2014). The European Rescue of the Washington Consensus? EU and IMF Lending to Central and Eastern European Countries. Review of International Political Economy 21(2): 310–338; Tsoukala, P. (2020), Post-Crisis Economic and Social Policy—Some Thoughts on Structural Reforms 2.0. In: F. Bignami (ed.), EU Law in Populist Times—Crises and Prospects, Cambridge: Cambridge University Press, 67–90.

21 See *supra* n. 16.

22 See Goldmann, M., ‘Are Fiscal Policy Rules Part of the Solution or Part of the Problem? On the False Dichotomy between Rules and Discretion’, Paper presented at ADEMU Conference (Madrid, 18–19 May 2018).

23 Dawson, M. (2015), ‘The Legal and Political Accountability Structure of Post-Crisis EU Economic Governance’. Journal of Common Market Studies 53(5): 981–983.

24 As an example, financial sanctions under the preventive and corrective arms of the SGP have been evoked and considered by EU institutions over the past decade, but were never concretely applied.

25 In this regard, see Martucci, F. (2018), La longue marche vers le cadre budgétaire intégré de la zone euro. Revue de l’Union Européenne 616: 160–163.

26 See for example Türk, A., Xanthoulis, N. (2019), ‘Legal accountability of European Central Bank in banking supervision—A case study in conceptualizing the legal effects of Union acts’. Maastricht Journal of Comparative and European Law 26(1): pp. 151–164; Molinari, C. (2019), ‘The EU and its perilous journey through the migration crisis—Informalzation of the EU return policy and rule of law concerns’. European Law Review: 824–840; Wessel, R. (2021), ‘Normative transformations in EU external relations—The phenomenon of ‘soft’ international agreements’. West European Politics 44(1): pp. 72–92.

27 In general, on the constitutional challenges raised by soft law, see Senden, L., *supra* n. 15, 477–497.

28 Discussing the issue of accountability and visibility in EU economic governance, Crum speaks of the ‘problem of many hands’, common when responsibility is shared between many actors while nobody can ultimately be held accountable. See, Crum, B. (2018), Making Democracy the Priority in EU Economic Governance—Four Theses on the Foundations of the T-DEM Project. European Papers 3(1): 63–64. Sacriste and Vauchez talk of a ‘puissant effet de brouillage’ (a powerful interference effect) between the national and the supranational levels (Sacriste, G., Vauchez, A. (2019), L’Euro-isation de l’Europe—Trajectoire historique d’une politique ‘hors les murs’ et nouvelle question démocratique. Revue de l’OFCE 165: 4).

29 Tridimas, T. (2018), Indeterminacy and legal uncertainty in EU law. In J. Mendes (ed.), EU Executive Discretion and the Limits of Law. Oxford: OUP, 40–63.

30 On soft law and competences, see De Witte, B. (2018), ‘The place of the OMC in the system of EU competences and sources of law. European Papers 3(1): pp. 207–213.

31 Most famously, non-privileged applicants must establish a direct and individual concern, a condition difficult to meet in practice. In general, see Lenaerts, K., Gutman, K., Maselis, I. (2014), EU Procedural Law. Oxford, OUP, 316–337.

32 Senden, L., Van Den Brink, T. (2012), Checks and Balances of Soft EU Rule-Making, Report for the European Parliament, PE 462.433, pp. 64–68.

33 In general, see Curtin, D. (2014), Challenging Executive Dominance in European Democracy. Modern Law Review 77(1): 1–32.

34 At best, the Parliament’s intervention is consultative. This is for example the case for the Semester CSR’s, which are discussed before the Parliament under the Economic Dialogue. On the limited involvement of the European Parliament in the Eurozone economic governance, see Crum, B. (2017), Parliamentary accountability in multi-level governance—What Role for Parliaments in EU Post-Crisis Economic Governance. Journal of European Public Policy 25(2): 268–286.

35 See Markakis, M. (2020), Accountability in the Economic and Monetary Union. Oxford: OUP, 105–151.

36 In general, on post-legislative guidance, see Scott, J. (2011), In Legal Limbo: Post-Legislative Guidance as a Challenge for European Administrative Law. Common Market Law Review 48(2): 329–355.

37 On that point, see however Court of Justice, judgment of 19 July 2016, C-356/14, *Kotnik*, § 40.

38 These inconsistencies are recurrently highlighted by the European Fiscal Board in its annual reports. For the latest edition, see EFB, Annual Report 2020, 28 September 2020.

39 Leino, P., Saarenheimo, T. (2018), Discretion, Economic Governance and the (New) Political Commission. In J. Mendes (ed.), *supra* n. 29, 132–154.

40 Among many others, see Abbott, K., Slaughter, A.-M, Moravcsik, A., Keohane, R. (2000), The Concept of Legalization. International Organizations 54(3): 401–419; Senden, L., supra n. 15; Terpan, F. (2015), Soft Law in the European Union—The Changing Nature of EU Law. European Law Journal 21(1): 68–96; Stefan, O. (2012), European Union Soft Law—New Developments concerning the Divide between Legally Binding Force and Legal Effects. Modern Law Review 75(5): 979–893; Eliantonio, M., Stefan, O. (2018), Soft Law Before the European Courts—Discovering a Common Pattern?. Yearbook of European Law 37(1): 457–469.

41 Most notably, see ECJ, judgment of 13 December 1988, C-322/88, *Grimaldi*.

42 It is primarily in the context of annulment actions based on Article 263 TFEU that this method was developed. A central condition to the admissibility of such action is indeed the existence of a challengeable act, a notion which was initially interpreted rather widely as covering all acts intended to produce legal effects (following the famous ‘ERTA formula’; Court of Justice, judgment of 31 March 1971, case C-22/70, *Commission v Council*, § 42), but which later on narrowed down to ‘acts producing binding legal effects’.

43 Gentile, G. (2020), Ensuring Effective Judicial Review of EU Soft Law via the Action for Annulment before the EU Courts—A Plea for a Liberal-Constitutional Approach. European Constitutional Law Review 16(3), pp. 14–18.

44 In that regard, see for example Court of Justice, judgment of 13 October 2011, case C-463/10 and C-475/10, *Deutsche Post and Germany v Commission*, § 36; Court of Justice, judgment of 25 October 2017, case C-599/15P, *Romania v Commission*, § 47; Court of Justice, judgment of 20 February 2018, case C-16/16P, *Kingdom of Belgium v Commission*, § 31.

45 For similar endeavors, see Stefan, O. (2014), Helping Loose Ends Meet? The Judicial Acknowledgement of Soft Law as a Tool of Multi-Level Governance. *Maastricht Journal of Comparative and European Law* 21(2): 359–379; Gentile, G., *supra* n. 44, pp. 1–20; Eliantonio, M. (2021), Judicial Review of Soft Law Before the European and the National Courts—A Wind of Change Blowing From the Member States?. In M. Eliantonio, E. Korkea-Aho, O. Stefan (eds), EU Soft Law in the Member States. Oxford: Hart.

46 This alternative approach to the divide between hard and soft law put forward in this article is sketched out in the specific context of Eurozone fiscal governance, but has the potential to be applied in other policy fields (not considered here) characterized by a similar disconnect.

47 Opinion AG Bobek, C-16/16P, *Kingdom of Belgium v Commission*. As a general comment, AG Bobek has been very active in calling the Court to revisit the key concepts governing its jurisdiction and the admissibility of the cases brought before it. See, on the concept of individual concern, see AG Bobek, C-352/19P, *Région de Bruxelles-Capitale v Commission*.

48 *Ibid.*, § 82.

49 *Ibid.*, § 88.

50 *Ibid.*, § 95.

51 *Ibid.*, § 108.

52 *Ibid.*, § 67.

53 See *ibid.*, § 75.

54 *Ibid.*, § 113.

55 *Ibid.*, §§ 115–122.

56 Their operative sections are generally preceded by a high number of recitals, revealing a certain level of complexity and maturity. A great majority of them are published in the Official Journal and featured in the C series. All of these instruments, even those of a country-specific nature (most notably, the country-specific recommendations), are available in all official languages of the EU.

57 Council recommendation of 9 July 2019 on the 2019 National Reform Programme of Finland and delivering a Council opinion on the 2019 Stability Programme of Finland, 5.9.2019, *OJ* C 301, pp. 154–159.

58 Council recommendation of 13 July 2018 on the 2018 National Reform Programme of Italy and delivering a Council opinion on the 2018 Stability Programme of Italy, 10.9.2018, *OJ* C 320, pp. 48–52.

59 Council of the European Union, Recommendation with a view to bringing an end to the situation of an excessive government deficit in Portugal, 18 June 2013, *OJ* C180, 26.6.2013, pp. 4–8.

60 On the importance of perception, see General Court, judgment of 4 March 2015, case T-496/11, *United Kingdom v European Central Bank*, esp. § 48. See also Xanthoulis, N. (2021), Legal Effects and Reviewability of EU Acts—Between Institutional Intentions and Perception of Concerned Parties. In M. Eliantonio, E. Korkea-Aho, O. Stefan (eds), EU Soft Law in the Member States. Oxford: Hart.

61 For example, Regulation No. 1466/97 states that Member States are to take due account of the guidance addressed to them in the framework of the European Semester and, most notably, of the CSRs (Article 2a(3)(2)). In a similar fashion, Regulation No. 473/2013 makes it clear that the guidance offered by the Commission in its opinions on the draft budgetary plans is to be ‘appropriately integrated’ in the national budgets (Recital 21 and Article 1(1)(c)).

62 Compliance with past CSRs constitutes a key factor in the framework of the opening of an EDP (as a relevant factor; see Article 126(3) TFEU), in the ‘significant deviation’ assessment under the preventive arm of the SGP (Article 6(2) of Regulation No. 1466/97) and in the Commission assessment of the DBP under Regulation No. 473/2013.

63 This ‘public communication’ aspect of certain EU acts was highlighted remarkably by AG Cruz Villalon in his *Gauweiler* opinion and played an important role in him finding that a press release announcing the OMT program constituted an EU act producing legal effects, the validity of which could thus be examined by the Court in the framework of proceedings for a preliminary ruling (Opinion of AG Cruz-Villalon, in Case C-62/14, *Gauweiler and Others v Deutscher Bundestag*, § 84–90). In a similar manner, the communicative power of its opinions under Regulation No. 473/2013, clearly participates in the Commission’s strategy in the field of fiscal coordination and surveillance and the same would hold for most key outputs of Eurozone fiscal governance.

